# Advances in Semiconductor Lasers Based on Parity–Time Symmetry

**DOI:** 10.3390/nano14070571

**Published:** 2024-03-26

**Authors:** Hongbo Sha, Yue Song, Yongyi Chen, Jishun Liu, Mengjie Shi, Zibo Wu, Hao Zhang, Li Qin, Lei Liang, Peng Jia, Cheng Qiu, Yuxin Lei, Yubing Wang, Yongqiang Ning, Guoqing Miao, Jinlong Zhang, Lijun Wang

**Affiliations:** 1 State Key Laboratory of Luminescence and Applications, Changchun Institute of Optics, Fine Mechanics and Physics, Chinese Academy of Sciences, Changchun 130033, China; 2 Daheng College, University of Chinese Academy of Sciences, Beijing 100049, China; 3 Jlight Semiconductor Technology Co., Ltd., Changchun 130033, China

**Keywords:** semiconductor lasers, parity–time (PT) symmetry, quantum mechanics, longitudinal modulation in PT-symmetric structures, transverse modulation, distributed-feedback laser, micro-ring/micro-disk lasers, Bragg reflective waveguide lasers, photonic crystal lasers

## Abstract

Semiconductor lasers, characterized by their high efficiency, small size, low weight, rich wavelength options, and direct electrical drive, have found widespread application in many fields, including military defense, medical aesthetics, industrial processing, and aerospace. The mode characteristics of lasers directly affect their output performance, including output power, beam quality, and spectral linewidth. Therefore, semiconductor lasers with high output power and beam quality are at the forefront of international research in semiconductor laser science. The novel parity–time (PT) symmetry mode-control method provides the ability to selectively modulate longitudinal modes to improve the spectral characteristics of lasers. Recently, it has gathered much attention for transverse modulation, enabling the output of fundamental transverse modes and improving the beam quality of lasers. This study begins with the basic principles of PT symmetry and provides a detailed introduction to the technical solutions and recent developments in single-mode semiconductor lasers based on PT symmetry. We categorize the different modulation methods, analyze their structures, and highlight their performance characteristics. Finally, this paper summarizes the research progress in PT-symmetric lasers and provides prospects for future development.

## 1. Introduction

The invention of semiconductor lasers significantly impacted the advancement of modern optical technology. Compared to other lasers, semiconductor lasers have a higher conversion efficiency, achieving efficiencies of up to 70% [1]. They are crucial for optical fiber communication, medical diagnostics, laser radars, sensors, and other applications. According to a recent analysis by Future Market Insights (FMI), the global semiconductor laser market is expected to reach CNY 8.8 billion by 2023, with a projected compound annual growth rate of 5.9% in revenue from 2023 to 2033 [2]. High-power, high-beam-quality semiconductor lasers are at the forefront of international research in semiconductor laser science [3]. Lasers are primarily classified according to their emission method, including vertical-cavity surface-emitting lasers (VCSELs) and edge-emitting lasers (EELs). VCSELs exhibit characteristics such as circular output beams, low threshold currents, and easy integration into large-area arrays [4]. They have widespread applications in optical communications, optical interconnects, and optical storage. In contrast, EELs exhibit superior output power and are widely used in laser processing, pumping of solid-state and fiber lasers, spectral analysis, and other applications [5]. As the scope of semiconductor lasers continues to expand, the demand for a higher output power is increasing. The traditional approach for increasing power output is to continuously improve the electro-optical conversion efficiency; however, this has reached its effective limit (about 70% [1]). Otherwise, larger injection currents are required to attain a higher output power; unfortunately, increasing the pump current while keeping the cross-sectional area fixed leads to an increased current density, triggering power-limiting effects such as nonlinear losses [6] and catastrophic optical damage (COD) [7]. This problem is most effectively solved by increasing the cross-sectional area of the laser, which increases the optical output power while maintaining the current density below the optical damage threshold. However, increasing the cross-sectional area may cause higher-order modes to emerge and reduce the beam quality.

In recent decades, extensive research has been conducted on semiconductor lasers, with a focus on realizing single-mode semiconductor lasers characterized by a small size, low threshold, high efficiency, and high side-mode suppression ratio (SMSR). To select longitudinal modes, traditional semiconductor lasers often employ structures such as distributed feedback (DFB) gratings [8,9], distributed Bragg reflector (DBR) gratings [10,11], and narrow slots [12] along the cavity length. Lateral mode selection can be achieved using various structures, including ridge waveguides [13,14], chirped arrays [15,16], and tilted cavities [17,18]. These methods have proved to be effective in improving laser performance. However, DFB and DBR lasers exhibit complex structures that increase process complexity and manufacturing costs, despite their ability to effectively select a single longitudinal mode; and, despite their ability to achieve single transverse mode operation, semiconductor lasers with narrow ridge waveguide structures struggle to achieve a high power, owing to their small current injection area.

Therefore, traditional semiconductor lasers often sacrifice other characteristics to control the optical field [19,20,21,22,23]. To achieve high-power, high-beam-quality, single-mode lasers, researchers have begun to explore quantum-mechanical concepts. The Schrödinger equation in quantum mechanics and the Helmholtz equation in optical systems have formal equivalence, making their solutions analogous. Therefore, concepts from quantum mechanics, such as parity–time (PT) symmetry, can be utilized to control laser optical fields, providing the possibility of attaining the desired laser-system features.

PT symmetry primarily addresses the real eigenvalue problem in open systems. By modulating the relationship between the creation and annihilation operators in an open system and its system parameters, the system can exhibit partially closed system properties, even when operating above exceptional points in the parameter space, where energy exchange with the external environment is maintained. By leveraging the properties of PT symmetry, the modulation of gain and loss (contrasting the creation and annihilation operators) in waveguides concerning the system parameters can lead to real or complex propagation constants for different transverse optical modes. This modulation allows certain optical modes to exist in degenerate states with PT symmetry—where the propagation constant becomes complex—resulting in gain or loss during the optical-mode propagation. Simultaneously, other optical modes exist in non-degenerate states under spontaneous symmetry breaking, resulting in real propagation constants, and thus propagating without gain or loss.

Therefore, by strategically placing the PT-symmetric exceptional points, the fundamental mode can operate above the exceptional points with the other modes operating below. This facilitates a clear distinction between the fundamental and higher-order modes. The introduction of PT symmetry offers a new avenue for optimizing the mode characteristics of semiconductor lasers, provides an additional method for mode control, and has emerged as a novel research direction in semiconductor laser science. Currently, PT symmetry is widespread in laser applications. For example, single transverse mode operation can be achieved by the lateral introduction of a PT-symmetric coupling structure into a laser [24], and the longitudinal introduction of PT-symmetric Bragg gratings into lasers allows for the selection of longitudinal modes [25,26]. Additionally, at the PT-symmetric breaking points, peculiar phenomena such as unidirectional light transmission [27,28] are observed. These unique characteristics can be exploited for coherent perfect absorber lasers (CPAL) and other applications [29,30,31,32,33].

Starting with the fundamental principles, this study introduces the background of PT symmetry, covering the transition from PT symmetry in quantum mechanics to its application in optical systems. We also explore the concept of PT symmetry breaking. Subsequently, two PT-symmetry modulation methods for lasers are discussed: lateral and longitudinal modulation. The developmental history and research progress of these two modulation methods are discussed. Finally, the advantages and limitations of the two methods are summarized.

## 2. Basic Principles

### 2.1. PT Symmetry in Quantum Mechanics

PT symmetry originates from quantum mechanics, where the stationary Schrödinger equation describing the motion of particles is given by

(1) Hψ=Eψ,

where H is the Hamiltonian operator describing the total energy of the system, ψ is the wave function corresponding to a stationary state, and *E* is the eigenvalue associated with the energy of the system. In quantum mechanics, because the energy of the system is an observable quantity and each observable operator must be Hermitian, the Hamiltonian operator must be Hermitian. This is because the eigenvalues of a Hermitian operator are real numbers.

It was not until 1998 that Bender et al., at the University of Washington [34] introduced a class of non-Hermitian Hamiltonians. If the PT symmetry condition is satisfied, then the system can possess real eigenvalues. In this case, the system is referred to as PT-symmetric.

PT symmetry requires that the PT-symmetric operator commutes with the Hamiltonian operator:

(2) $$\left[PT,H\right]=PTH-HPT=0.$$

Here, the operators P and T correspond to the spatial inversion and time reversal operators, respectively, with specific actions defined as follows:

(3) $$P\psi \left(x\right)=\psi \left(-x\right),$$

(4) $$T\psi \left(x\right)=\psi^{*}\left(x\right).$$

Applying these operators to the Hamiltonian operator results in the following:

(5) $$PTH=\frac{{\hat{p}}^{2}}{2m}+V^{*}\left(x\right),$$

(6) $$HPT=\frac{{\hat{p}}^{2}}{2m}+V\left(x\right),$$

where ${\hat{p}}^{2}$ is the momentum operator, m is the system mass, and $V\left(x\right)$ is the potential function of the system. Therefore, if the Hamiltonian operator satisfies the PT symmetry condition, it must satisfy Equation (2).

(7) $$V\left(x\right)=V^{*}\left(-x\right)$$

Equation (7) implies that a Hamiltonian operator satisfying PT symmetry has a potential function with even symmetry, in terms of spatial coordinates, for its real part and odd symmetry for its imaginary part. Bender et al., also found that there is a threshold [35,36,37,38,39,40] for the imaginary part of the potential function of the Hamiltonian that satisfies PT symmetry. When the imaginary part is smaller than the real part, the energy eigenvalues are real and the system is in a PT-symmetric state. Conversely, when the imaginary part is larger than the real part, the energy eigenvalues are complex and the system is in a PT-symmetry-broken state. This threshold is known as the PT-symmetry exceptional point [41].

### 2.2. PT symmetry and PT-Symmetry Breaking in Optical Systems

In optical systems, the Helmholtz equation describes the electromagnetic field and is given by the following:

(8) $$\nabla^{2}\overset{⃑}{E}+k^{2}\bar{\varepsilon }\left(\overset{⃑}{x}\right)\overset{⃑}{E}=0,$$

where k represents the wave vector and $\bar{\varepsilon }\left(\overset{⃑}{x}\right)$ is the position-dependent relative permittivity of the material. A comparison of the Helmholtz equation with the stationary Schrödinger equation shows that the two equations are identical in form. In this context, the quantum mechanical potential term corresponds to the permittivity of an optical system. Therefore, for an optical system to satisfy PT symmetry, the permittivity or refractive index should satisfy the following conditions:

(9) $$\bar{\varepsilon }\left(\overset{⃑}{x}\right)={\bar{\varepsilon }}^{*}\left(-\overset{⃑}{x}\right),n\left(\overset{⃑}{x}\right)=n^{*}\left(-\overset{⃑}{x}\right).$$

In other words, the real part of the material’s permittivity or refractive index should exhibit even symmetry concerning the spatial coordinates, whereas the imaginary part should display odd symmetry. Because the imaginary part of the refractive index represents the gain or loss in the material, the introduction of gain and loss is necessary to satisfy PT symmetry in an optical system. This modulation involves simultaneous adjustments of both the real and imaginary parts of the refractive index of the material. Structurally, this modulation can be categorized into two types: longitudinal modulation along the direction of light propagation, and transverse modulation perpendicular to the direction of light propagation.

Similar to the breaking of PT symmetry in quantum mechanics, there is also a threshold point in optical systems. This occurs when the modulation of the imaginary part of the refractive index exceeds that of the real part, causing the optical system to enter a state of PT-symmetry breaking. This results in changes in the optical behavior, such as unidirectional invisibility caused by unidirectional loss and asymmetric reflection spectra owing to the direction-dependent incidence of light, as has been demonstrated both theoretically and experimentally [27,42]. These characteristics are utilized in applications such as optical switches [43,44], PT-symmetric lasers [25,45], coherent perfect absorbers [29,46], and optical logic [47]. 

#### 2.2.1. Longitudinal Modulation in PT-Symmetric Structures

A PT-symmetric Bragg grating is the most common application of PT-symmetric longitudinal modulation. In this type of grating, the refractive index varies periodically along the propagation direction, which enhances the interaction between the forward- and backward-propagating waves. The interference and superposition of these two waves results in high reflectivity at specific frequencies, known as the Bragg frequency. This occurs under the Bragg condition:

(10) $$f_{B}=\frac{c_{0}}{2n_{avg}\Lambda }.$$

The Bragg frequency $f_{B}$ depends on the physical periodic length (Λ) of the grating and the average refractive index ($n_{avg}$) of the grating, where $c_{0}$ represents the speed of light in a vacuum. In practice, the grating strength reflects the width and slope of the transmission and reflection spectra. It is determined by the number of periods (N) of the grating and the modulation depth ($\Delta n^{\prime }$) of the refractive index.

Introducing a refractive-index distribution with PT symmetry into a Bragg grating results in a PT-symmetric Bragg grating structure. The PT symmetry in the refractive-index distribution requires the real part of the refractive index to be an even function, whereas the imaginary part (representing the gain and loss) must be an odd function. Consequently, the refractive index of a PT-symmetric Bragg grating can be expressed as follows:

(11) $$n=\left(n_{avg}\pm \Delta n^{\prime }\right)\pm i\Delta n^{\prime \prime },$$

where $n_{avg}$ represents the average refractive index, and $\Delta n^{\prime }$ and $\Delta n^{\prime \prime }$ denote the modulation depths of the real and imaginary parts of the refractive index, respectively. When designing this PT-symmetric Bragg grating to operate above an exceptional point, the eigenstates of the structure become degenerate. In such a scenario, the reflection from one side of the grating is amplified, whereas the reflection from the other side is attenuated. However, the transmitted light remains unaffected by the grating, and its transmission coefficient and phase remain unchanged. In 2011, Lin et al., from Wesleyan University in the United States [27] first reported a PT-symmetry-broken Bragg grating that demonstrated unidirectional invisibility when operating near the PT-symmetric exceptional point. 

#### 2.2.2. Transverse Modulation in PT-Symmetric Structures

The simplest structure for PT-symmetric transverse modulation is a PT-symmetric coupled waveguide [48]. The two waveguides have identical propagation constants, but different gain and loss characteristics. With low gain or loss, the coupled-mode theory provides an effective approximation of the amplitude variation of the system modes. Under such circumstances, the coupled-mode equations governing the amplitude variations in the system are expressed as follows:

(12) $$-i\frac{d}{d\xi }\left(\begin{array}{c} a\\ b \end{array}\right)=\left(\begin{array}{cc} \delta +i\gamma & \kappa \\ \kappa & \delta -i\gamma \end{array}\right)\left(\begin{array}{c} a\\ b \end{array}\right),$$

where δ represents the propagation constant in an individual waveguide, ±γ denotes the distributed gain/loss constants, and κ represents the coupling coefficient between the two waveguides. The variable ξ represents the longitudinal coordinate z for an optical waveguide, or the time t in the case of an optical cavity. Suppose the mode takes the form: $\left(a,b\right)=\left(A,B\right)e^{i\Omega \xi }$; in this case, Equation (12) can be simplified as follows:

(13) $$\left(\begin{array}{cc} \delta +i\gamma & \kappa \\ \kappa & \delta -i\gamma \end{array}\right)\left(\begin{array}{c} a\\ b \end{array}\right)=\Omega \left(\begin{array}{c} a\\ b \end{array}\right).$$

The corresponding Hamiltonian is given by the following:

(14) $$H=\left(\begin{array}{cc} \delta +i\gamma & \kappa \\ \kappa & \delta -i\gamma \end{array}\right).$$

This Hamiltonian operator is not Hermitian, because $H^{†}\neq H$; however, it is a PT-symmetric operator. According to the definitions of the parity operator (P) and time operator (T)

(15) $$P\left(\begin{array}{c} A\\ B \end{array}\right)=\left(\begin{array}{c} B\\ A \end{array}\right),$$

(16) $$T\left(\begin{array}{c} A\\ B \end{array}\right)=\left(\begin{array}{c} A^{*}\\ B^{*} \end{array}\right).$$

It was readily demonstrated that H satisfies the necessary conditions for PT symmetry: $\left[PT,H\right]=PTH-HPT=0$. Therefore, as long as PT symmetry is preserved, the non-Hermitian operator has real eigenvalues. Thus, the eigenvalues in Equation (13) fall into two cases; the first case is when the coupling coefficient is greater than the contrast between the gain and loss (κ>γ):

(17) $$\Omega_{1,2}=\delta \pm \sqrt{\kappa^{2}-\gamma^{2}},$$

(18) $$\left(\begin{array}{c} A_{1,2}\\ B_{1,2} \end{array}\right)=\left(\begin{array}{c} 1\\ -i\gamma /\kappa \pm \sqrt{1-(\gamma /\kappa {)}^{2}} \end{array}\right).$$

Here, the two supermodes are symmetrically distributed between the two channels, and the intensities of the two modes are equal $|A_{1,2}{|}^{2}=|B_{1,2}{|}^{2}$.

If the coupling coefficient is less than the contrast between the gain and loss (κ<γ), PT symmetry breaks and the eigenvalues are no longer real but take the form of complex conjugates:

(19) $$\Omega_{1,2}=\delta \pm i\sqrt{\gamma^{2}-\kappa^{2}},$$

(20) $$\left(\begin{array}{c} A_{1,2}\\ B_{1,2} \end{array}\right)=\left(\begin{array}{c} 1\\ -\frac{i\gamma }{\kappa }+i\sqrt{(\gamma /\kappa {)}^{2}-1} \end{array}\right).$$

When the PT symmetry is broken, the symmetry between the two modes disappears. At the critical point where the transition between PT symmetry and PT-symmetry breaking occurs (κ=γ), the two eigenvalues become degenerate:

(21) $$\Omega_{1,2}=\delta ,$$

(22) $$\left(\begin{array}{c} A_{1,2}\\ B_{1,2} \end{array}\right)=\left(\begin{array}{c} 1\\ i \end{array}\right).$$

At this point, both eigenvalues and eigenvectors undergo degeneracy [49]. Beyond this exceptional point, the real parts of the two eigenvalues are equal, whereas the imaginary parts are opposite.

Thus, the optical system is in a PT-symmetric state when the gain–loss contrast is smaller than the coupling coefficient. The two modes are symmetrically distributed between the two channels with equal intensities. As the gain or loss increases, the separation between the two modes gradually decreases until it reaches zero when the gain–loss contrast equals the coupling coefficient. By further increasing the gain or loss, when the gain–loss contrast exceeds the coupling coefficient, the real parts of the two modes become equal and the imaginary parts become opposite. This indicates that, at a specific frequency, one mode in one waveguide experiences gain, whereas the mode in the other waveguide experiences loss.

## 3. Research Progress on Longitudinal Modulation in PT-Symmetric Systems

PT-symmetric longitudinal modulation involves refractive-index modulation parallel to the direction of light propagation, allowing for the selection of longitudinal modes. By controlling the intensity of the gain or loss, the desired mode can be placed in a PT-symmetry-broken state while the other modes remain in a PT-symmetric state, thus filtering out the desired longitudinal mode. In less than a decade since the first theoretical proof of transmission asymmetry in longitudinally modulated PT-symmetric Bragg gratings for applications in optically pumped lasers and, more recently, in electrically pumped lasers, this field has developed rapidly. PT-symmetric structures have found widespread application in lasers owing to their excellent mode-selection characteristics. Based on their different PT-symmetric structures, they can be classified into four main types: DFB, micro-ring/micro-disk, CPAL, and photonic crystal lasers. Table 1 summarizes the research progress on transverse PT-symmetric modulation.

### 3.1. PT-Symmetric DFB Lasers

PT-symmetric DFB lasers are constructed using PT-symmetric Bragg gratings instead of traditional refractive-index-modulated gratings. By exploiting the transmission asymmetry of PT-symmetric gratings, the spatial hole burning effects, mode competition, and mode hopping problems associated with traditional single-mode DFB lasers that require a λ/4 phase shift grating [9,50] or other complex structures [51,52,53] are avoided. This approach effectively achieves the single-mode characteristics of DFB lasers.

In 2011, Lin et al., from Wesleyan University [27] first theoretically demonstrated the transmission asymmetry of longitudinally modulated Bragg gratings based on PT symmetry. Moreover, they demonstrated that such gratings exhibit unidirectional invisibility during the breaking of PT symmetry. Their study lays a theoretical foundation for the realization of single-mode lasers based on PT-symmetric longitudinal modulation.

**Figure 1:**
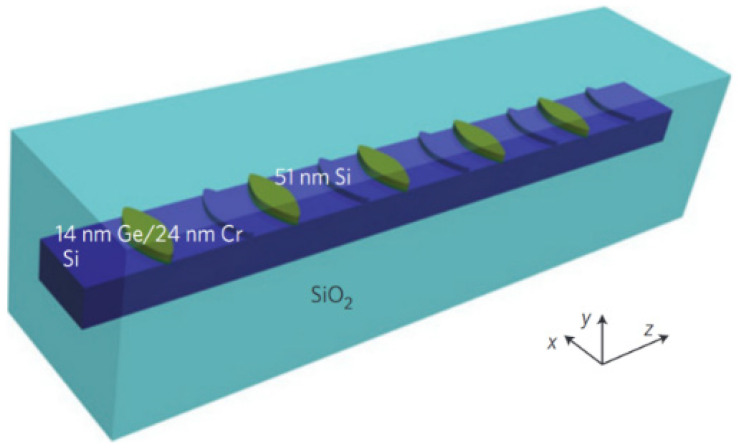
PT-symmetric optical structures. Periodically arranged 760 nm-wide sinusoidal-shaped combo structures are applied on top of an 800 nm-wide Si waveguide embedded inside SiO_2_ to mimic parity–time optical potentials, in which imaginary part modulation is implemented with 14 nm Ge/24 nm Cr structures and 51 nm Si layers are for real part modulation. Reprinted with permission from Ref. [28]. Copyright 2013, Springer Nature.

In 2013, Feng et al., from the University of Florida [28] experimentally observed asymmetric transmission in longitudinal PT-symmetric structures for the first time. The structure shown in Figure 1 was fabricated using electron-beam lithography and dry etching to form Si waveguides. To achieve these sinusoidal-function modulated optical potentials, sinusoidal-shaped combo structures (a combination of a sinusoidal-shaped structure and its mirror image to the transverse direction) are adopted on top of the Si waveguide. The real and imaginary modulation of the refractive index is realized by additional Si and Cr/Ge bilayer combination structures, respectively. Optical coupling to the waveguide was achieved using optical fibers, and forward and backward reflectivities were measured. The experimental results showed that the forward reflectivity was 7.5 dB higher than the backward reflectivity, which agrees with the theoretical simulation results and confirms the optical asymmetry of the PT-symmetric structure. This further demonstrates the feasibility of laser experiments based on the PT-symmetric structure.

**Figure 2:**
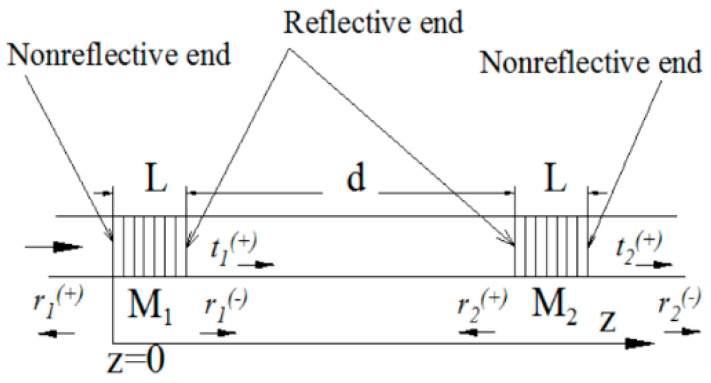
Schematic of a Fabry–Perot cavity composed of dual gratings; M1 and M2 are PT-symmetric unidirectional gratings and non-reflective ends placed in the cavity outwards. Reprinted with permission from Ref. [54]. Copyright 2013, Optical Society of America.

In 2013, Kulishov et al., from HTA Photomask [54] pioneered the simulation and design of DFB/DBR structures using PT-symmetric Bragg gratings. The grating structure achieved unidirectional light propagation at 1550 nm through periodic phase and amplitude modulations. As shown in Figure 2, the structure consisted of two unidirectional Bragg gratings connected to the reflective end, facing inward, to form a resonant cavity. Unlike conventional DFB/DBR lasers, light could enter the cavity entirely from one end, rather than being reflected. In addition, this PT-symmetric structure supported single-mode laser output, providing a new approach for realizing single-mode DFB/DBR lasers.

In 2016, Zhu et al., from Clemson University [55] conducted a simulation-based comparison of the mode characteristics of DFB structures with PT-symmetric gratings and conventional DFB structures. They found that DFB lasers with PT-symmetric gratings exhibited improved mode-discrimination capabilities, resulting in superior single-mode characteristics. First, they used the transfer matrix method to analyze the mode characteristics of PT-symmetric DFB lasers with a grating period of 500 nm, a real-part refractive index of 1.55, and a central wavelength of 1550 nm. The threshold gain for different laser modes was compared between traditional DFB lasers and those with PT-symmetric structures. The threshold gain for the lowest-loss mode in PT-symmetric DFB lasers was 35.30 cm^−1^, lower than that of conventional DFB lasers. The threshold gain difference between the lowest-loss mode and the second-order mode increased from 13.70 cm^−1^ in traditional DFB architecture to 36.57 cm^−1^ in PT-symmetric DFB structures. This trend was also observed in DFB structures with λ/4 phase shifts, and the phenomenon became more pronounced with an increasing imaginary-part refractive index. Thus, the PT-symmetric DFB structures exhibited lower threshold gains and higher mode-discrimination capabilities. This suggests that PT-symmetric gratings improve the single-mode stability of DFB lasers. The study emphasizes the advantages of PT-symmetric lasers over conventional lasers and demonstrates their application potential.

In 2017, Ke et al., from the Huazhong University of Science and Technology [56] proposed an external optical feedback DFB laser that utilized PT-symmetric complex coupling. The grating exhibited strong asymmetric reflection of the counter-propagating light within the DFB cavity, owing to the PT symmetry of the complex refractive index. This effectively blocked the return light from one end. The sensitivity of the DFB laser to the external optical feedback was significantly reduced. Numerical simulations were used to compare the laser wavelength and SMSR of traditional gain-coupled and refractive-index-coupled DFB lasers with varying external optical feedback. The simulations were compared with those of a DFB laser incorporating PT-symmetric structures. The study showed that the PT-symmetric DFB laser had a wavelength variation of only 0.2 nm with external feedback at −10 dB, and the SMSR remained above 45 dB. The PT-symmetric DFB laser demonstrated superior resistance to interference compared to other DFB laser structures.

**Figure 3:**
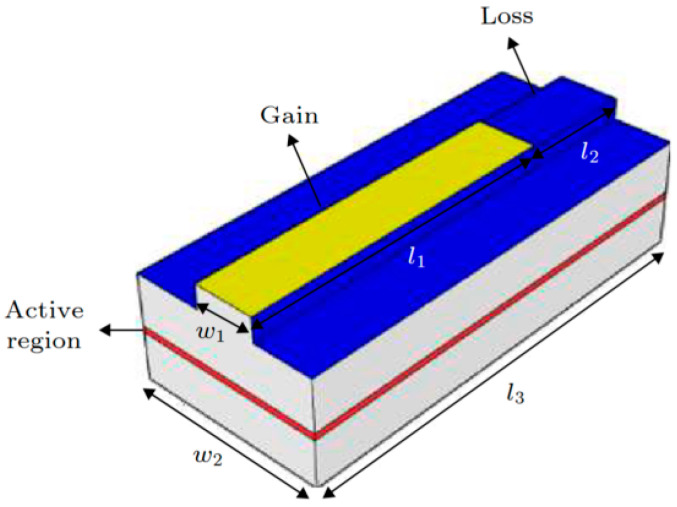
Schematic of an electrically injected PT-symmetric laser structure. The waveguide layer consists of Al(x)GaAs, which has a refractive index of n ≈ 3.42. The quantum well is made up of GaAs and GaIn(x)As. Reprinted with permission from Ref. [26]. Copyright 2020, Science Press.

In 2020, Wang et al., at the Institute of Semiconductors of the Chinese Academy of Sciences [26] introduced the concept of PT symmetry in electrically injected semiconductor lasers with stripe structures. They analyzed the impact of the gain–loss structure symmetry on the PT symmetry-breaking phenomenon and demonstrated experimentally that PT symmetry breaking could lead to a doubling of the mode spacing in electrically injected semiconductor lasers. Figure 3 shows a schematic of this structure. The simulation and analysis initially focused on the effects of different loss-region losses on the PT symmetry-breaking threshold. The results showed that as the losses increased, the gain required for PT symmetry breaking decreased, and the mode degeneracy near the breaking point increased. The impact of different ratios between the gain and loss regions on the PT symmetry-breaking threshold point was further analyzed. The results showed that a smaller loss-region proportion leads to a higher gain requirement in the gain region to break PT symmetry. Finally, the PT symmetry-breaking phenomenon was experimentally observed in electrically injected semiconductor lasers. After reaching the threshold for the PT symmetry current, mode degeneracy and doubling of the mode spacing were observed.

**Figure 4:**
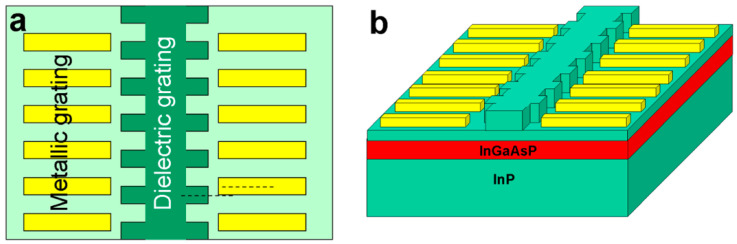
(**a**) Principle of PT-symmetric gratings, with symmetry planes of gratings (dashed lines) shifted by a quarter. (**b**) Schematic of a DFB laser structure with PT-symmetric grating. The waveguide layer consists of InP and the quantum well is made up of InGaAsP. Reprinted with permission from Ref. [45]. Copyright 2021, De Gruyter.

In 2021, de la Perrière et al., of the Center for Nanosciences and Nanotechnologies in France [45,57] experimentally realized a DFB laser based on PT-symmetric dual gratings, with a central wavelength of 1550 nm. The laser structure used lateral ripple gratings to modulate the real part of the refractive index and deposited lateral metal stripes to modulate the imaginary part of the refractive index, as shown in Figure 4. 

In their study, several key PT-symmetric DFB laser performance parameters, including the output power, spectrum, and transmission characteristics were compared to those of conventional DFB lasers. The PT-symmetric DFB laser demonstrated an output threshold of 40 mA, reaching 16 mW per facet at 200 mA with a SMSR exceeding 50 dB. The study also made comparisons to pure refractive-index-coupled and pure gain-coupled lasers. At 120 mA, the pure refractive-index-coupled DFB exhibited multimode behavior, whereas the third-order pure gain-coupled DFB barely reached the laser threshold. Therefore, the combination of the refractive index and gain coupling proved beneficial in enhancing the emission power and single-mode performance. The study further compared the results with different phase shifts for real and imaginary modulations and concluded that better performance was achieved under PT-symmetric conditions. The study demonstrated that this characteristic arises from unidirectional transmission, which mitigates the impact of reflected light on the overall optical field modes. The performance was comparable to that of traditional first-order grating devices using third-order dielectric and absorbing gratings, further confirming the advantages and development potential of PT-symmetric structures.

### 3.2. PT-Symmetric Micro-Ring/Micro-Disk Lasers

A PT-symmetric micro-ring laser is operated by manipulating the gain and loss of the micro-ring resonator to confine the desired mode to the gain region. Simultaneously, the gain and loss for the other modes are balanced, resulting in the emission of a single-mode laser light. Compared to conventional micro-ring lasers, this structure is not constrained by the bandwidth limitations of the gain spectrum, which inherently supports the generation of single-mode lasers.

**Figure 5:**
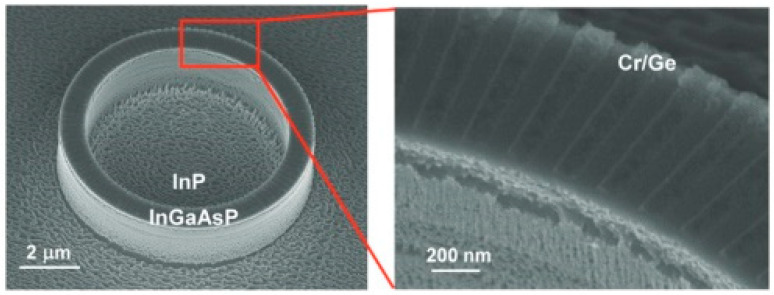
Scanning electron microscope image of PT-symmetric micro-ring laser. © The American Association for the Advancement of Science. Reprinted with permission from Ref. [25]. Copyright 2014, The American Association for the Advancement of Science.

In 2014, Feng et al., from the University of Florida [25] experimentally achieved the first PT-symmetric micro-ring laser operating in the 1500 nm wavelength range. The laser used a purely imaginary-part modulation. The structure was manufactured by growing InGaAsP quantum-well layers on an InP substrate and introducing periodic Cr/Ge modulation to the quantum-well layers to achieve periodic gain and loss distributions. Simultaneously, the impact of these materials on the real part of the refractive index was minimal (on the order of 0.01%). This resulted in a threshold-less breaking of the PT symmetry, allowing only the modes meeting specific conditions to experience a net gain and produce single-mode laser emission. In contrast to traditional micro-ring lasers with multiple competing modes, PT-symmetric micro-ring lasers can realize a single-mode output under arbitrary gain spectrum widths. Traditional lasers rely on the modulation of the real part of the refractive index for mode selection, which is limited by the bandwidth of the gain spectrum. Figure 5 shows scanning electron microscopy images of the structure. Photoluminescence was observed at 1500 nm for the first time when pumped using femtosecond lasers. As the pump power increased, a single-mode laser peak was observed at 1513 nm, with a SMSR exceeding 14 dB. Compared to traditional micro-ring lasers without additional gain modulation, the introduction of PT-symmetric gain-loss modulation did not alter the mode distribution within the micro-ring resonator, but effectively selected the desired mode. This groundbreaking work achieved a single-mode output without being limited by the gain spectrum bandwidth, providing a solid foundation for subsequent developments in PT-symmetric single-mode lasers.

In 2015, Song et al., from the Harbin Institute of Technology [58] presented a simulated design for a PT-symmetric micro-disk laser with single-mode characteristics and directional emission. A PT-symmetric structure was achieved by introducing one-dimensional periodic gain and loss into a circular micro-disk. They designed the micro-disk with a radius of 5 μm and a grating period of 1 μm, with a real-part refractive index of 1.56. An appropriate imaginary refractive index was chosen to position the first-order mode above the PT-symmetry-breaking threshold while keeping the other modes below the threshold. This resulted in a single-mode output at 919.8 nm. In addition, the periodic gain–loss modulation acted as a grating, enabling vertical distribution and achieving horizontal directional emission with a divergence angle of approximately 34° when the imaginary refractive index was 0.009. Compared to traditional lasers modulated along the azimuthal angle, this one-dimensional gain–loss modulation grating is easier to implement and offers directional output, showing potential for on-chip single-mode micro-disk lasers.

**Figure 6:**
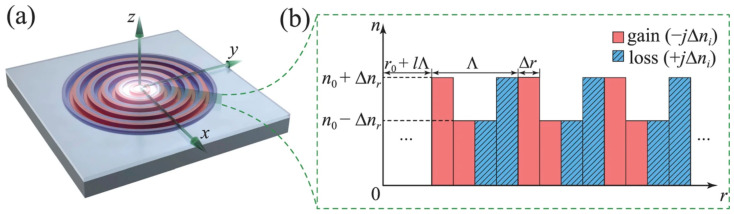
(**a**) Schematic of the PT-symmetric circular Bragg laser. (**b**) Distribution of the refractive index in the radial direction, showing periodic modulation of both the real and imaginary parts of the refractive index, where $n_{0}$ is the average effective refractive index. $\Delta n_{r}$ and $\Delta n_{i}$ are the modulation depths of the real and imaginary parts of the refractive index, respectively. Reprinted with permission from Ref. [59]. Copyright 2014, Springer Nature.

In 2016, Gu et al., from the Chinese University of Hong Kong [59] introduced PT symmetry into the design of a circular Bragg laser by implementing PT-symmetric structures in a two-dimensional circular Bragg grating. This innovation achieved a single-mode output at 1550 nm, where both the real and imaginary parts of the refractive index were radially modulated. Figure 6a illustrates the two-dimensional PT-symmetric circular Bragg laser on a chip. The PT symmetry is obtained in the circular Bragg reflector by introducing modulation to both the real and imaginary parts of the refractive index along the radial direction (r), as shown in Figure 6b. The mode characteristics of the laser were analyzed using the transfer matrix method and the results were validated using numerical simulations based on the actual design. The analysis revealed that a negative threshold gain occurred when the real and imaginary parts were simultaneously modulated. This implies that laser emission can be achieved without additional gain, even in the presence of losses in the central disk region. In contrast, traditional lasers that only modulate the real part of the refractive index require a positive threshold gain, regardless of the modulation depth. Therefore, compared to traditional lasers with only real-part modulation, PT-symmetric circular Bragg lasers exhibit significantly lower thresholds and enhanced mode-discrimination capability. This characteristic contributes significantly to the development of high-power single-mode lasers with mode-hop-free operation.

In 2017, Zhu et al., of Clemson University [60] analyzed the mode characteristics of two different types of PT-symmetric micro-ring lasers using the transfer matrix method. The first type utilized gain/loss modulation in the micro-ring laser, whereas the second type incorporated refractive-index/gain/loss modulation. This study found that PT-symmetric micro-ring lasers have a higher mode-discrimination capability than traditional micro-ring resonators. This is true even when quasi-symmetric gain/loss distributions, unbalanced perturbation amplitudes, and duty cycles exist. The PT-symmetric micro-ring laser exhibited stable single-mode characteristics, showing enhanced mode-discrimination capabilities over traditional micro-ring resonators.

### 3.3. PT-Symmetry-Based Coherent Perfect Absorber Lasers

A CPAL based on PT symmetry uses PT symmetry breaking to create coexisting resonant modes with identical frequencies in the cavity, encompassing both lasing and absorbing modes. Coherent amplification or absorption can be achieved using a single device by precisely manipulating the phase of the input light.

In 2010, Longhi of Politecnico di Milano [32] introduced the concept of CPAL and demonstrated that lasers meeting PT-symmetry conditions in their dielectric constants could exhibit the characteristics of a laser oscillator and a coherent perfect absorber. This novel class of lasers is referred to as PT-symmetric CPAL.

**Figure 7:**
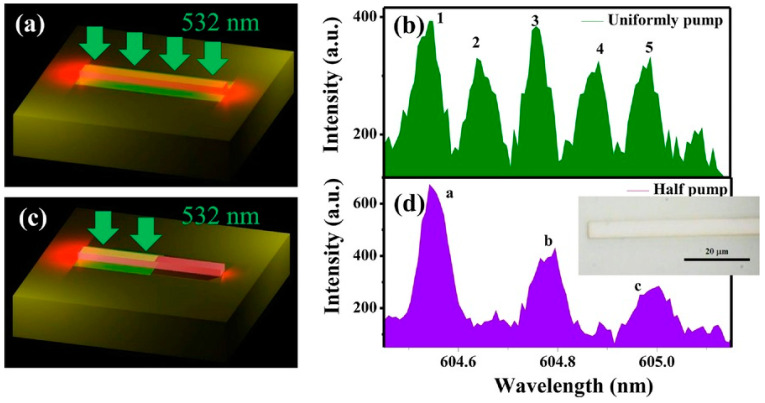
Laser spectra under (**a**,**b**) full and (**c**,**d**) half pumping. (**b**,**d**) are their corresponding laser spectra. Here, the pumping power is 23 μJ. The inset in (**d**) is the microscopic image of the sample. The width and length of the waveguide are 5 μm and 1 mm, respectively. Reprinted with permission from Ref. [61]. Copyright 2016, John Wiley and Sons.

In 2016, Gu et al., from the Harbin Institute of Technology [61] conducted research on PT-symmetric stripe lasers, which resulted in the first experimental confirmation of PT-symmetric CPAL. The stripe waveguide used had a width of 5 μm and a total length of 1 mm. Symmetric gain and loss were achieved by pumping half of the waveguide. Figure 7 shows the laser spectra under full- and half-pumping conditions. A comparison between the spectra under full pumping and half pumping was conducted with a pumping power of 23 μJ and wavelength of 532 nm. The results show that at around 605 nm, the number of spectral modes under half pumping was reduced by half and the mode spacing was doubled compared to full pumping. This led to the emergence of gain and loss modes with identical wavelengths, which is consistent with theoretical predictions and effectively confirms the breaking of PT symmetry. This work is significant for investigating the physical phenomena near the PT-symmetric threshold and for practical applications of lasers based on PT symmetry.

**Figure 8:**
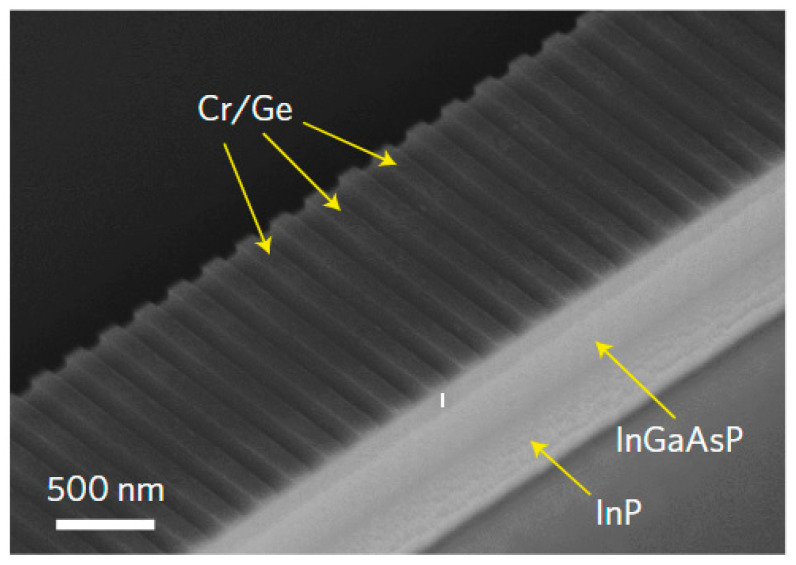
Image of the CPAL manufactured on an InGaAsP/InP substrate. Reprinted with permission from Ref. [62]. Copyright 2016, Springer Nature.

In 2016, Wong et al., from the University of California [62] successfully fabricated a PT-symmetric CPAL that experimentally realized lasing and antialiasing at the same frequency within a single cavity. The CPAL shown in Figure 8 was fabricated on an InGaAsP/InP substrate. It comprised two multi-mode interferometers (MMI) and a PT-symmetric CPAL. The longitudinal PT-symmetric structure, which allows pure gain–loss modulation, was achieved by growing InGaAsP quantum wells on an InP substrate, followed by the periodic deposition of Cr/Ge. Under a pump power of 4.2 µJ·cm^−2^, single-mode lasing with a PT-symmetry-breaking phase was achieved. The successful excitation of the intrinsic anti-lasing mode, which is mainly localized to the loss region, was achieved by restricting the phase shift between two probe beams to π/2. This clearly demonstrates coherent amplification and absorption with symmetric amplitudes at the same wavelength, confirming the coexistence of the laser and anti-laser modes in the CPAL. The PT-symmetric concept confirmed the existence of these modes. This CPAL has a range of potential applications as a laser, amplifier, modulator, and absorber, and incorporates numerous useful on-chip optical functionalities. It has further potential for use in photonic integrated circuits and offers a significant reduction in structural complexity.

### 3.4. PT-Symmetric Vertical Bragg Reflective Waveguide Lasers

A PT-symmetric vertical Bragg reflective waveguide (BRW) laser enhances the lateral gain by introducing additional mode modulation in the vertical direction through PT-symmetric Bragg gratings. This structure reduces the longitudinal cavity length, enlarges the mode spacing of the FP modes, and achieves a higher SMSR.

In 2018, Peng et al., from Shandong University [63] proposed and analyzed a single-mode edge-emitting laser based on a PT-symmetric vertical BRW structure. This configuration enhances the vertical mode gain, shortens the longitudinal cavity length, increases the FP mode spacing, and improves the SMSR. The mode-selection mechanism of the PT-symmetric Bragg reflective waveguide laser was analyzed to investigate the influence of key design parameters on laser performance, such as the number of Bragg grating layers, cavity length, and complex refractive-index perturbation. This study achieved a single-mode laser output with a high threshold gain difference, low threshold current, high slope efficiency, and high SMSR. By optimizing the structural parameters, a mode spacing of 13 nm and SMSR of more than 30 dB were achieved. This PT-BRW structure demonstrates the potential for high-power electrically pumped single-mode edge-emitting lasers through structural optimization.

In 2022, Peng et al., from Shandong Jianzhu University [64] proposed a vertical BRW laser, conducted simulations, and achieved a single-mode output at the PT-symmetry-breaking point. The structure had a mode spacing of 14 nm and a gain difference of 2.1 cm^−1^, which was sufficient to achieve 30 dB SMSR.

This configuration produced a single-mode laser output with a high SMSR and slope efficiency under an electrical injection of 1.6 mA. Compared to conventional edge-emitting lasers, this structure introduced gain on both sides of the cladding and suppressed the central core layer modes. Under PT-symmetry-breaking conditions, this design realized a stable single-mode laser while reducing the cavity length, increasing the mode spacing, and achieving a high SMSR.

### 3.5. PT-Symmetric Photonic Crystal Lasers

PT-symmetric photonic crystal lasers use a uniform pumping method to achieve a gain–loss distribution by eliminating air holes in the photonic crystal.

In 2022, Wang et al., from Zhejiang University [65] proposed a PT-symmetric photonic crystal laser with a single-mode output. They used a uniform pumping method and modulated the gain and loss by removing air holes from the photonic crystal cavity to realize a single-mode output. Without compromising the pump power and linewidth, they achieved a SMSR of 30 dB for a single-mode laser output. In addition, the output power of the desired mode was six times that of the multi-mode laser output. This approach provides a potential solution for stable single-mode lasing and high laser output power in devices based on nanophotonic structures.

This technique can also be used to control the mode properties of lasers by utilizing the mode field separation characteristics of the PT-symmetric phases in longitudinal PT-symmetric systems. Additionally, the unidirectional invisibility of beams at the PT-symmetry-breaking points can be exploited to realize orbital angular momentum (OAM) microcavity lasers [66,67]. The introduction of PT-symmetric longitudinal modulation into semiconductor lasers provides many solutions for improving performance; however, it has limitations owing to the need to introduce gain/loss to achieve PT symmetry, which can be challenging when the precise control of the gain/loss values is required. Currently, studies on PT-symmetric longitudinal modulation are primarily based on theoretical simulations. However, recent advancements in fabrication technologies have led to additional experimental studies. Many new structures have been explored and the performance of the devices continues to improve [68,69,70]. As technology continues to evolve, PT symmetry is expected to become more widely applied in semiconductor lasers.

**Table 1:** Research progress on PT-symmetric longitudinal modulation.

Year	Institution	Structure	Materials of Active Region	Wavelength	SMSR	Output Power	Simulation/ Experiment	References
2014	University of Florida	PT-symmetric micro-ring lasers	InGaAsP	1513 nm	14 dB	-	Experiment	[25]
2016	Harbin Institute of Technology	PT-symmetry-based coherent perfect absorber lasers	-	604 nm	-	-	Experiment	[61]
2017	Huazhong University of Science and Technology	PT-symmetric DFB lasers	-	-	45 dB	-	Simulation	[56]
2018	Shandong University	PT-symmetric vertical Bragg reflective waveguide lasers	InAlGaAs	1550 nm	30 dB	-	Simulation	[63]
2021	Center for Nanosciences and Nanotechnologies in France	PT-symmetric DFB lasers	InGaAsP	1550 nm	50 dB	16mW	Experiment	[45]
2022	Shandong Jianzhu University	PT-symmetric vertical Bragg reflective waveguide lasers	InGaAsP	1310 nm	30 dB	-	Simulation	[64]

## 4. Research Progress on Transverse Modulation in PT-Symmetric Systems

PT-symmetric transverse modulation involves the modulation of the refractive index perpendicular to the direction of light propagation. The introduction of transverse PT-symmetric structures allows the output power of a laser to deviate from a monotonic change with increasing gain or loss. For example, an increase in gain may lead to a decrease in laser intensity [71], whereas an increase in loss can induce laser suppression and recovery [72]. Transverse PT symmetry allows for the selection of transverse modes by exploiting the different coupling coefficients of various modes. By controlling the intensities of the gain and loss and appropriately selecting the PT symmetry-breaking threshold, it is possible to achieve a single transverse mode output, which enhances the beam quality and mode characteristics of the laser. Additionally, transverse PT-symmetric dual micro-ring-coupled lasers can be used to filter the longitudinal modes. Table 2 summarizes the research progress on transverse PT-symmetric modulation, which, in terms of structure, can be classified into PT-symmetric coupled waveguide structures and PT-symmetric coupled micro-ring structures. In terms of pumping methods, the transverse modulation of PT symmetry mainly goes through two stages: the optical pumping stage and electrical pumping stage. Typically, due to the impacts of carrier injection and doping, the threshold of electrically pumped structures is slightly elevated in comparison to optically pumped structures. Furthermore, in the structural design of optically pumped lasers, there is no necessity to account for conductive structures or carrier concentration, with their output performance being predominantly influenced by the pump source area and pump power. However, when contrasted with electrically pumped structures, optically pumped structures exhibit a lower degree of integration. Electrically pumped structures, being directly driven by electrical current, achieve a higher level of integration. 

### 4.1. Lasers Based on PT-Symmetric Coupled Waveguide Structures

A PT-symmetric coupled waveguide comprises gain and loss waveguides. By controlling the contrast between gain and loss, high-order modes are confined to below the PT symmetry-breaking threshold, with only the fundamental mode existing in the PT-symmetry-breaking phase. Consequently, the fundamental mode propagates exclusively in the gain waveguide, whereas the other modes are uniformly distributed between the gain and loss waveguides. This configuration improves the beam quality.

In 2010, Rüter et al., from the Clausthal University of Technology [73] observed asymmetry in wave propagation in a laterally modulated PT-symmetric structure. They used Fe-doped LiNbO_3_ as a coupled waveguide, modulating the real part of the refractive index through Ti doping, and providing gain through nonlinear refractive-index modulation via two-wave mixing. A pump-light area controlled using a mask allowed for the amplification of only one waveguide, resulting in a PT-symmetric coupled waveguide structure. As the gain increases, the wave propagation becomes asymmetric. Once the threshold was surpassed, one mode was amplified, whereas the other mode was attenuated, which is consistent with the numerical simulation results. This experimental study validated the asymmetry of wave propagation with nonreciprocal propagation in PT-symmetric coupled waveguides, providing avenues for the development of novel optical devices.

In 2012, Miri et al., from the University of Central Florida [74] proposed a method for realizing large-area single-mode lasers. They introduced the concept of PT-symmetric transverse modulation into the laser design, positioning the fundamental mode above the PT-symmetry-breaking point, while situating the other modes below it. Consequently, this method amplified only the fundamental mode, resulting in a large-area single-mode output—the concept of breaking PT symmetry is the theoretical foundation of realizing large-area single-mode output lasers.

**Figure 9:**
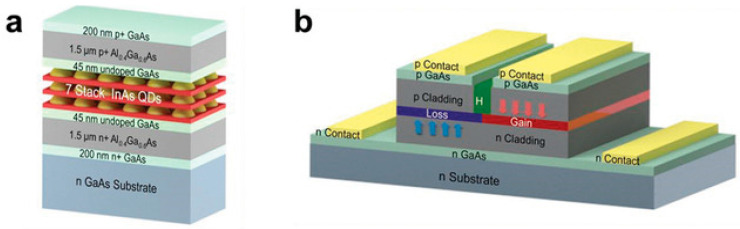
(**a**) Heterostructure and (**b**) InAs quantum dot PT-symmetric coupled waveguide laser. Reprinted with permission from Ref. [75]. Copyright 2019, John Wiley and Sons.

In 2019, Yao et al., from the University of Massachusetts [75] designed and fabricated a PT-symmetric coupled waveguide laser with independent electrically tunable gain and loss, as shown in Figure 9. Electrically pumped devices offer more effective control over the gain and loss in the waveguide than optically pumped devices. This laser exhibited a single transverse mode and a broad area. By independently tuning the gain and loss in the two waveguides through electric injection, higher-order transverse modes were suppressed, resulting in a single transverse mode output. Furthermore, the wide-area coupled waveguide laser produced an approximately single-lobed far-field mode. The study successfully demonstrated a PT-symmetric coupled waveguide laser under electrical injection, opening new possibilities for the practical application of PT symmetry in optics.

In 2021, Fu et al., from the Institute of Semiconductors of the Chinese Academy of Sciences [76] fabricated a PT-symmetric dual-ridge semiconductor laser with a central wavelength of 980 nm and characterized its properties. The structural schematic consists of two ridges with widths of 5 μm and a separation of 2 μm. The electrical injection window was located on one side of the ridge. The ridge depth was 1 μm and the cavity length was 6 μm. The PT-symmetric dual-ridge waveguide laser had a narrower linewidth than traditional dual-ridge waveguide lasers. At 91 mA, the SMSR of the PT-symmetric laser reached 37.97 dB. In terms of the far-field distribution, the PT-symmetric laser operates in the PT-symmetric phase at lower currents, where both modes have equal gains. As the current increases, the system enters the PT symmetry-breaking phase, in which one mode obtains a larger gain and the other mode receives a smaller gain, resulting in a single-lobed far-field distribution. The introduction of PT symmetry improves the beam quality and enhances the mode characteristics of the laser. 

In 2021, Yang et al., from the Huazhong University of Science and Technology [77] proposed and demonstrated a PT-symmetric electrically injected single-mode FP laser. This PT-symmetric laser comprised two identical FP resonators that allowed control over gain and loss through the adjustment of the injection current. The single-mode operation was achieved by selectively breaking the PT symmetry. The PT-symmetric laser structure achieved a single-mode laser output at 1330 nm with an output power of 1.7 dBm and a side-mode suppression ratio exceeding 24 dB. In addition, when the system is in a PT-symmetry-broken state, it exhibits a single-lobed far-field mode similar to that of a single-ridge laser. Therefore, the PT-symmetric structure enabled the realization of a high-quality single-mode output while increasing the injected current area.

**Figure 10:**
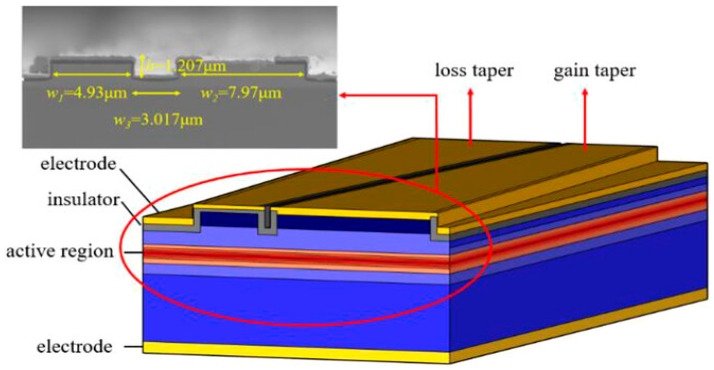
Schematic and SEM of a biconical PT symmetric semiconductor laser. Reprinted with permission from Ref. [78]. Copyright 2022, John Wiley and Sons.

In 2022, Wang et al., of the Institute of Semiconductors of the Chinese Academy of Sciences [78] fabricated a PT-symmetric double-cone semiconductor laser. The laser structure is shown in Figure 10. To obtain gain, the insulating layer of one cone cavity was removed and covered with an electrode through inductively coupled plasma (ICP) etching, whereas the other cone cavity was insulated to serve as a lossy cone, achieving a PT-symmetric laser structure. By reducing the PT symmetry-breaking point below the threshold, the laser achieved an output power exceeding 500 mW with a current injection of 0.3 A. Compared to traditional dual-ridge lasers and single-ridge lasers, the PT-symmetric double-cone laser not only effectively suppressed lasing in higher-order transverse modes but also reduced the number of longitudinal modes, resulting in a narrower linewidth. In addition, the far-field distribution exhibited a single-lobed pattern. 

**Figure 11:**
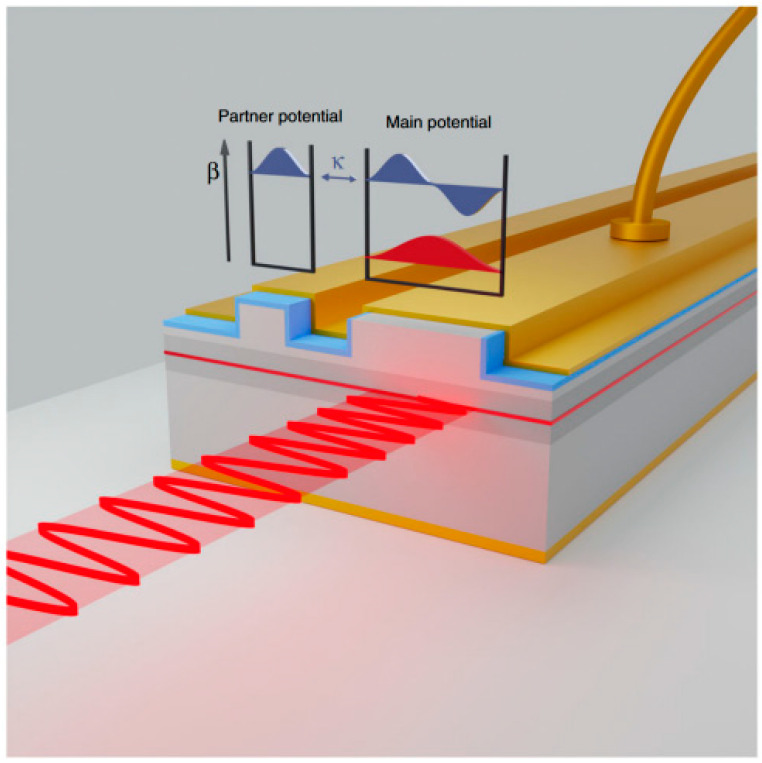
Schematic of a quasi-PT symmetric laser. The concept of quasi-PT symmetric edge-emitting lasers is realized with detuned coupled waveguide cavities. The propagation constant β decreases with increasing mode number, while the mode coupling is denoted by κ. By carefully selecting the cavity dimensions for isospectral engineering, resonant interactions between the second-order modes of the main potential and the fundamental modes of its lossy partners can be achieved, thus establishing quasi or modal PT symmetry. In this design, the electrical pumping of the main waveguide can act as a mode filter, enabling single-mode laser emission. Reprinted with permission from Ref. [79]. Copyright 2023, Springer Nature.

In 2023, Seker et al., from the Institute of Materials Science and Nanotechnology at Bilkent University [79] developed an electrically pumped, large-area, edge-emitting quasi-PT-symmetric laser that achieved a high-power single-mode output, as shown in Figure 11. This addresses the issue of low beam quality caused by higher-order modes in large-area lasers. The quasi-PT-symmetric structure was achieved by introducing an accompanying waveguide. The second-order modes in the main and accompanying waveguides were the same. Consequently, the second-order mode in the main waveguide could propagate in both waveguides, whereas the fundamental mode could only propagate in the main waveguide. Selective pumping in the main waveguide was performed through electrical injection to boost the gain of the fundamental mode and suppress the higher-order mode outputs, resulting in single transverse mode lasing. The laser achieved a high beam quality (M^2^ = 1.25) and a high-power output of 400 mW, whereas conventional lasers have an output power of 50 to 100 mW [80,81]. This study demonstrated that introducing PT symmetry into laser structures can result in high output power and excellent beam quality.

In 2023, Yang et al., from the Huazhong University of Science and Technology [82] achieved a single transverse mode output using a multiridge PT-symmetric laser array. They independently adjusted the gain and loss in the two laser arrays by employing two separate P-side electrodes to achieve PT-symmetrical or PT-symmetry-breaking states. The multiridge coupled waveguide array enhanced the coupling coefficient, increasing the gain difference between the fundamental and second-order modes. This addresses the issues associated with poor beam quality in wide-area coupled waveguides caused by low coupling coefficients and small gain differences that lead to mode competition. At the same current density, the emission power of the four-ridge PT-symmetric laser array was approximately five times higher than that of a single-ridge laser. Furthermore, this structure maintained single transverse mode characteristics over a ridge-spacing range of 1.06–1.42 μm, exhibiting a large process tolerance. This study extended PT-symmetric coupled waveguides to an array structure, which is significant for designing single-mode, wide-area, high-output-power lasers.

### 4.2. Lasers Based on PT-Symmetric Coupled Micro-Ring Structures

PT-symmetric coupled micro-rings utilize the unique threshold gains and coupling coefficients of various modes. By placing gain and loss micro-rings adjacent to each other, the modes of the two micro-rings are coupled. The desired mode is selected by controlling the intensities of the gain and loss in the two micro-rings. Compared to traditional micro-ring lasers that achieve single-mode output through gain control, PT-symmetric coupled micro-ring structures exhibit higher mode-discrimination capabilities.

**Figure 12:**
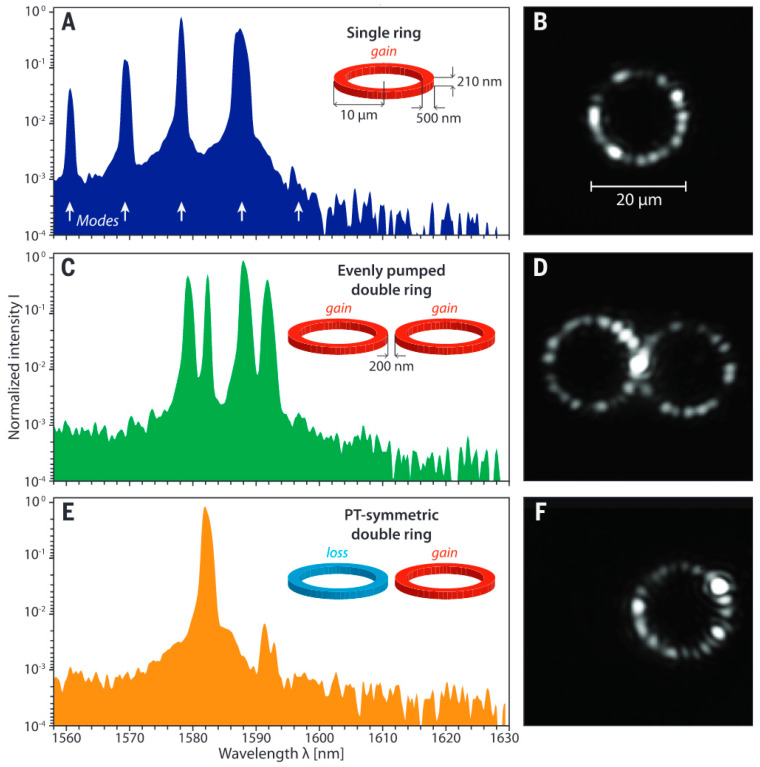
Experimental demonstration of PT-symmetry-breaking phase mode suppression. (**A**) Emission spectrum of a single resonator (radius 10 mm, ring width 500 nm, height 210 nm) when exposed to a peak pump power of 4.9 mW. (**B**) Corresponding intensity pattern in the ring observed from scattered light. (**C**) Emission spectrum obtained from a pair of uniformly pumped rings spaced 200 nm apart (4.9 mW + 4.9 mW). (**D**) Both rings have the same pattern intensity. (**E**) Single-mode spectrum under PT-symmetric conditions (0 mW + 4.9 mW pump). The SMSR exceeds 20 dB. (**F**) Lasing exclusively occurs in the active resonator. Reprinted with permission from Ref. [83]. Copyright 2014, The American Association for the Advancement of Science.

In 2014, Hodaei et al., from the University of Central Florida [83] designed a laser with a coupled micro-ring structure. They achieved single-mode laser output through PT-symmetric lateral modulation. The structure comprised two micro-rings with a radius of 10 μm, width of 500 nm, and thickness of 210 nm, spaced 200 nm apart. Optical pumping was applied to one of the micro-rings, causing the gain and loss to be distributed in opposite directions. In this scenario, the modes are divided into gain and loss modes, enabling mode selection. This structure achieved a single-mode laser output at 1580 nm with an optical pumping power of 4.9 mW and a SMSR exceeding 20 dB. Traditional methods can also achieve single-mode operation by controlling the gain; however, the gain usually does not surpass the threshold difference between adjacent modes. In contrast to traditional approaches, the introduction of PT symmetry allows for a greater net gain in single-mode operation. This improvement is particularly significant when the difference in the gain threshold between adjacent modes is small, as shown in Figure 12. This study shows that PT-symmetric structures can improve the single-mode features of micro-ring resonators, eliminate multi-mode lasing, and enable the future development of PT-symmetric coupled micro-rings. 

In 2015, Hodaei et al., from the University of Central Florida [84] investigated the characteristics of coupled micro-rings at PT-symmetric exceptional points. They validated the theoretical framework near the exceptional points and achieved a continuous optical tuning of 3.3 nm. The coupled micro-ring structure consisted of two micro-rings with a radius of 10 μm, width of 500 nm, and thickness of 210 nm, separated by 150 nm. The spectral characteristics of the coupled micro-ring were investigated under nonuniform pumping conditions. Mode splitting occurs because of the coupling between the micro-rings under uniform pumping. The gap in the mode splitting gradually decreased as the loss in one of the micro-rings increased. When the difference between the loss and gain equaled the coupling coefficient, PT symmetry reached an exceptional point, resulting in single-mode lasing, which is consistent with the theoretical analysis. The tunability of PT-symmetric lasers by temperature was investigated using a coupled micro-ring structure. The spectral characteristics of the PT-symmetric micro-ring structure were observed at temperatures ranging from 270 to 300 K under different pumping powers. The wavelength tuning was found to be approximately 0.1 nm/K. Compared to a single micro-ring, the PT-symmetric mode showed stable self-adjustment characteristics, which significantly reduced mode hopping. The mode was continuously tuned over a range of 3.3 nm as the temperature varied from 270 K to 300 K.

**Figure 13:**
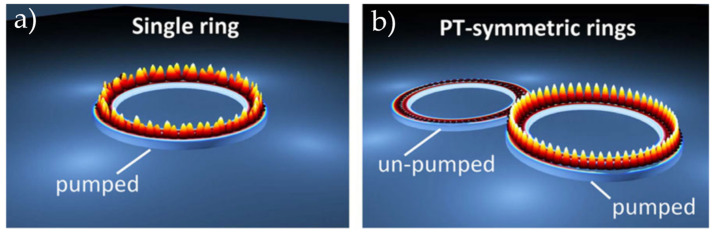
Schematic of a multimode single micro-ring and PT symmetric micro-rings. (**a**) In an isolated micro-ring resonator, multiple transverse and longitudinal modes can generate laser light simultaneously. (**b**) In a PT-symmetric arrangement, only one longitudinal mode with the lowest order transverse profile can emit laser light. Reprinted with permission from Ref. [24]. Copyright 2016, John Wiley and Sons.

In 2016, Hodaei et al., from the University of Central Florida [24] demonstrated that PT symmetry could facilitate the realization of the fundamental transverse mode in multi-mode micro-ring lasers, enabling single-mode lasing, as shown in Figure 13. A micro-ring structure was grown on an InP substrate with multiple InGaAsP quantum wells. It formed a circular resonator with a radius of 6 μm and waveguide dimensions of 0.21 μm × 1.5 μm. The PT-symmetric micro-ring coupling principle results in a lower fundamental-mode coupling coefficient than higher-order modes. Thus, the fundamental mode can achieve sufficient gain to break the PT symmetry and form a single mode. Unlike traditional independent micro-ring resonators, which exhibit multi-mode lasing owing to the closely spaced laser thresholds of several modes, PT-symmetric coupled micro-ring lasers suppress other modes, achieving a SMSR of up to 25 dB near 1587 nm with a linewidth of 10 GHz. Additionally, the slope efficiency of the PT-symmetric distribution was similar to that of the independent micro-rings. The PT-symmetric distribution can guide all power into a single mode, which is a significant improvement over independent micro-ring lasers. This method of suppressing transverse modes does not require the introduction of losses for other modes but instead maintains the desired mode in the active region, distributing other modes uniformly between gain and loss. Therefore, this approach provides superior mode-discrimination capabilities and enhances laser efficiency [83].

In 2017, Liu et al., from the University of Ottawa [85] proposed and experimentally demonstrated an electrically pumped PT-symmetric micro-ring laser. The laser achieved a high SMSR and single-mode tunable laser output. The PT-symmetric laser consisted of two mutually coupled micro-ring resonators. Precise control of the gain and loss was achieved by integrating multiple semiconductor optical amplifiers in the micro-ring resonators, satisfying the PT-symmetry conditions, or reaching PT-symmetry-breaking states for mode selection. Additionally, continuous-wavelength tuning was achieved by incorporating a phase modulator within the resonator. The result was a single-mode laser with a central wavelength at 1554.148 nm, featuring a SMSR exceeding 36 dB and continuous tuning in the range of 1553.800–1554.020 nm.

In 2017, Hayenga et al., of the University of Central Florida [86] proposed an electrically pumped PT-symmetric coupled micro-ring laser. The laser achieved a single-mode output, without affecting the output power or threshold current, through the interaction between the gain and loss. The laser structure consists of two micro-rings, each with a radius of 10 μm, width of 1 μm, and separated by 300 nm. The active layer consisted of InGaAsP with a thickness of 300 nm. The spectra of both the non-PT-symmetric and PT-symmetry-broken coupled micro-ring lasers indicated the presence of multiple longitudinal modes when both rings were uniformly pumped. When a current was applied to only one of the rings, the other ring experienced a loss, creating a PT-symmetry-breaking state. In this scenario, only the fundamental mode exhibits a significant SMSR, and the laser mode is exclusively present in the micro-ring with electrical pumping. 

In 2020, Hayenga et al. from the University of Central Florida [87] fabricated an electrically pumped micro-ring laser based on PT symmetry. This was the first realization of a microscale, electrically pumped single-mode PT-symmetric system without secondary growth within III-V semiconductor materials. The laser achieved a low threshold current of 445 μA near 1534 nm and a SMSR exceeding 18 dB for single-mode output. The device exhibited a slope efficiency identical to that of a uniformly pumped micro-ring laser. The implementation of an electrically pumped PT-symmetric laser enables on-chip light sources and modulators for PT-coupled micro-ring laser applications.

Compared to PT-symmetric longitudinal modulation, lateral modulation is easier to implement experimentally. This is because it does not require the fabrication of complex grating structures. Longitudinal modes can be selected using coupled micro-ring structures, whereas coupled waveguide structures can generate a single transverse mode. However, the filtering capability of longitudinal modes in coupled waveguide structures is relatively weak. Therefore, additional structures must be introduced to select specific longitudinal modes. With the development of technology, the PT-symmetric structure of the transverse modulation transitions from the optical to the electrical pumping stage and its single-mode characteristics have been gradually improved [88].

**Table 2:** Experimental research progress on PT-symmetric transverse modulation.

Year	Institution	Structure	Materials of Active Region	Wavelength	SMSR	References
2014	University of Central Florida	Optically pumped coupled micro-ring (1064 nm pumped)	InGaAsP	1580 nm	20 dB	[83]
2016	University of Central Florida	Optically pumped coupled micro-ring (1064 nm pumped)	InGaAsP	1587 nm	25 dB	[24]
2017	University of Ottawa	Electrically injected coupled micro-ring	InGaAsP	1554 nm	36 dB	[85]
2020	University of Central Florida	Electrically injected coupled micro-ring	InGaAs	1534 nm	18 dB	[87]
2021	Institute of Semiconductors of the Chinese Academy of Sciences	Electrically injected coupled waveguide	-	980 nm	37.97 dB	[76]
2021	Huazhong University of Science and Technology	Electrically injected coupled waveguide	-	1330 nm	24 dB	[77]

## 5. Conclusions and Outlook

Since its initial proposal by Bender et al., in 1998 [34], the concept of PT symmetry has been gradually applied in optics. It has emerged as a novel method for mode control and has become a focus of research in recent years. This paper provided a detailed overview of the technical approaches and current developments in single-mode semiconductor lasers based on PT symmetry, starting with its basic principles. The discussion can be divided into two main categories: longitudinal and lateral modulations. PT-symmetric lasers offer several advantages over traditional grating structures in terms of longitudinal modulation. They avoid spatial hole burning effects and the issue of multi-mode lasing caused by refractive-index gratings, allowing for the realization of single-mode lasing. PT-symmetric structures have been used in various lasers, including DFB, micro-ring/micro-disk, CPAL, and photonic crystal lasers. DFB lasers based on PT-symmetric structures have achieved a single-mode output with a SMSR exceeding 50 dB [45]. PT-symmetric laser structures for lateral modulation are simple and require only the addition of a lossy waveguide to achieve a single transverse mode output. This configuration enhances the laser beam quality. In PT-symmetric coupled waveguide lasers, a high beam quality with an M^2^ value of 1.25 and a high output power of 400 mW have been achieved [79]. Furthermore, PT-symmetric lateral modulation has been applied in coupled micro-ring structures.

The introduction of PT-symmetric structures has simplified semiconductor laser designs, improving both mode characteristics and spectral properties. However, longitudinal-modulation PT-symmetric lasers currently have high threshold currents and low output power, owing to the introduction of losses. However, advancements in fabrication technologies have the potential to make significant contributions to areas such as optical fiber and free-space optical communications. Additionally, lateral-modulation PT-symmetric laser structures are simple to implement and are expected to enable the development of high-power lasers with excellent beam quality in the future. These lasers have potential applications in laser processing, solid-state lasers, optical fiber laser pumping sources, spectral analysis, and other fields.

## References

[1] Yamagata Y., Kaifuchi Y., Nogawa R., Yoshida K., Morohashi R., Yamaguchi M. (2020). Highly efficient 9xx-nm band single emitter laser diodes optimized for high output power operation. Proc. SPIE.

[2] Future Market Insights. https://www.futuremarketinsights.com/reports/semiconductor-lasers-market.

[3] Wang L., Chen Y., Gao S., Guo L., Jia P., Lei Y., Liang L., Lv W., Peng H., Qin L. (2022). High Power, High Beam Quality Semiconductor Laser.

[4] Zhang J., Li X., Zhang J., Ning Y., Wang L. (2020). Research progress of vertical-cavity surface-emitting laser. Chin. J. Lumin..

[5] Chen L., Yang G., Liu Y. (2020). Development of Semiconductor Lasers. Chin. J. Lasers.

[6] Wenzel H., Crump P., Pietrzak A., Wang X., Erbert G., Tränkle G. (2010). Theoretical and experimental investigations of the limits to the maximum output power of laser diodes. New J. Phys..

[7] Tomm J.W., Ziegler M., Hempel M., Elsaesser T. (2011). Mechanisms and fast kinetics of the catastrophic optical damage (COD) in GaAs-based diode lasers. Laser Photonics Rev..

[8] Nakamura M., Yariv A., Yen H.W., Somekh S., Garvin H.L. (1973). Optically pumped GaAs surface laser with corrugation feedback. Appl. Phys. Lett..

[9] Usami M., Akiba S., Utaka K. (1987). Asymmetric λ/4-shifted InGaAsP/InP DFB lasers. IEEE J. Quantum Electron..

[10] Spießberger S., Schiemangk M., Wicht A., Wenzel H., Erbert G., Tränkle G. (2011). DBR laser diodes emitting near 1064 nm with a narrow intrinsic linewidth of 2 kHz. Appl. Phys. B.

[11] Müller A., Fricke J., Brox O., Erbert G., Sumpf B. (2016). Increased diffraction efficiencies of DBR gratings in diode lasers with adiabatic ridge waveguides. Semicond. Sci. Technol..

[12] Guo W.-H., Lu Q., Nawrocka M., Abdullaev A., O’Callaghan J., Lynch M., Weldon V., Donegan J.F. (2012). Integrable slotted single-mode lasers. IEEE Photonics Technol. Lett..

[13] Wenzel H., Bugge F., Dallmer M., Dittmar F., Fricke J., Hasler K.H., Erbert G. (2008). Fundamental-lateral mode stabilized high-power ridge-waveguide lasers with a low beam divergence. IEEE Photonics Technol. Lett..

[14] Miah M.J., Kettler T., Posilovic K., Kalosha V.P., Skoczowsky D., Rosales R., Bimberg D., Pohl J., Weyers M. (2014). 1.9 W continuous-wave single transverse mode emission from 1060 nm edge-emitting lasers with vertically extended lasing area. Appl. Phys. Lett..

[15] Kapon E., Lindsey C., Katz J., Margalit S., Yariv A. (1984). Chirped arrays of diode lasers for supermode control. Appl. Phys. Lett..

[16] Lindsey C.P., Kapon E., Katz J., Margalit S., Yariv A. (1984). Single contact tailored gain phased array of semiconductor lasers. Appl. Phys. Lett..

[17] DeMars S.D., Dzurko K.M., Lang R.J., Welch D.F., Scifres D.R., Hardy A. Angled-grating distributed feedback laser with 1 W cw single-mode diffraction-limited output at 980 nm. Proceedings of the Conference on Lasers and Electro-Optics.

[18] Sarangan A.M., Wright M.W., Marciante J.R., Bossert D.J. (1999). Spectral properties of angled-grating high-power semiconductor lasers. IEEE J. Quantum Electron..

[19] Liu L., Zhang J., Wang Y., Jiang B., Qu H., Zhang Y., Zheng W. (2012). 500-mW CW single-lobe emission from laterally coupled photonic crystal laser arrays. IEEE Photonics Technol. Lett..

[20] Liu L., Qu H., Wang Y., Liu Y., Zhang Y., Zheng W. (2014). High-brightness single-mode double-tapered laser diodes with laterally coupled high-order surface grating. Opt. Lett..

[21] Sun W., Lu Q., Guo W., Wallace M., Bello F., Donegan J.F. (2017). Analysis of high-order slotted surface gratings by the 2-D finite-difference time-domain method. J. Lightwave Technol..

[22] Ma X., Liu A., Qu H., Qi A., Zhou X., Wang M., Zheng W. (2018). High-power tapered photonic crystal lasers with slots for narrow spectral width. IEEE Photonics Technol. Lett..

[23] Dan L., Qiu Y., Hao W., Yi H., He Q., Huan W., Ling Z., Wei W. (2020). Review of semiconductor distributed feedback lasers in the optical communication band. Chin. J. Lasers.

[24] Hodaei H., Miri M., Hassan A.U., Hayenga W.E., Heinrich M., Christodoulides D.N., Khajavikhan M. (2016). Single mode lasing in transversely multi-moded PT-symmetric microring resonators. Laser Photonics Rev..

[25] Feng L., Wong Z.J., Ma R.M., Wang Y., Zhang X. (2014). Single-mode laser by parity-time symmetry breaking. Science.

[26] Wang X.-Y., Wang Y.-F., Zheng W.-H. (2020). Mode control of electrically injected semiconductor laser with parity-time symmetry. Acta Phys. Sin..

[27] Lin Z., Ramezani H., Eichelkraut T., Kottos T., Cao H., Christodoulides D.N. (2011). Unidirectional invisibility induced by PT -Symmetric periodic structures. Phys. Rev. Lett..

[28] Feng L., Xu Y.L., Fegadolli W.S., Lu M.H., Oliveira J.E., Almeida V.R., Chen Y.F., Scherer A. (2013). Experimental demonstration of a unidirectional reflectionless parity-time metamaterial at optical frequencies. Nat. Mater..

[29] Chong Y.D., Ge L., Cao H., Stone A.D., Stone A.D. (2010). Coherent perfect absorbers: Time-reversed lasers. Phys. Rev. Lett..

[30] Ge L., Chong Y.D., Rotter S., Türeci H.E., Stone A.D. (2011). Unconventional modes in lasers with spatially varying gain and loss. Phys. Rev. A.

[31] Chong Y.D., Ge L., Stone A.D. (2011). PT-symmetry breaking and laser-absorber modes in optical scattering systems. Phys. Rev. Lett..

[32] Longhi S. (2010). PT-symmetric laser absorber. Phys. Rev. A.

[33] Yang M., Ye Z., Zhu L., Farhat M., Chen P.-Y. (2022). Recent advances in coherent perfect absorber-lasers and their future applications. J. Central S. Univ..

[34] Bender C.M., Boettcher S. (1998). Real spectra in non-hermitian Hamiltonians having P T symmetry. Phys. Rev. Lett..

[35] Liertzer M., Ge L., Cerjan A., Stone A.D., Türeci H.E., Rotter S. (2012). Pump-induced exceptional points in lasers. Phys. Rev. Lett..

[36] Chen W., Kaya Özdemir Ş.K., Zhao G., Wiersig J., Yang L. (2017). Exceptional points enhance sensing in an optical microcavity. Nature.

[37] Kominis Y., Choquette K.D., Bountis A., Kovanis V. (2018). Exceptional points in two dissimilar coupled diode lasers. Appl. Phys. Lett..

[38] Hodaei H., Hassan A.U., Wittek S., Garcia-Gracia H., El-Ganainy R., Christodoulides D.N., Khajavikhan M. (2017). Enhanced sensitivity at higher-order exceptional points. Nature.

[39] Miri M.A., Alù A. (2019). Exceptional points in optics and photonics. Science.

[40] Özdemir Ş.K., Rotter S., Nori F., Yang L. (2019). Parity–time symmetry and exceptional points in photonics. Nat. Mater..

[41] Bender C.M., Brody D.C., Jones H.F. (2002). Complex extension of quantum mechanics. Phys. Rev. Lett..

[42] Kulishov M., Laniel J.M., Bélanger N., Azaña J., Plant D.V. (2005). Nonreciprocal waveguide Bragg gratings. Opt. Express.

[43] Nazari F., Nazari M., Moravvej-Farshi M.K. (2011). A 2×2 spatial optical switch based on PT-symmetry. Opt. Lett..

[44] Phang S., Vukovic A., Susanto H., Benson T.M., Sewell P. (2013). Ultrafast optical switching using parity–time symmetric Bragg gratings. J. Opt. Soc. Am. B.

[45] Brac de la Perrière V.B., Gaimard Q., Benisty H., Ramdane A., Lupu A. (2021). Electrically injected parity-time symmetric distributed feedback laser diodes (DFB) for telecom applications. Nanophotonics.

[46] Sun Y., Tan W., Li H.Q., Li J., Chen H. (2014). Experimental demonstration of a coherent perfect absorber with PT phase transition. Phys. Rev. Lett..

[47] Phang S., Vukovic A., Benson T.M., Susanto H., Sewell P. (2015). A versatile all-optical parity-time signal processing device using a Bragg grating induced using positive and negative Kerr-nonlinearity. Opt. Quantum Electron..

[48] Christodoulides D.N., Miri M.-A. (2014). PT symmetry in optics and photonics. Proc. SPIE.

[49] Heiss W.D. (2000). Repulsion of resonance states and exceptional points. Phys. Rev. E Stat. Phys. Plasmas Fluids Relat. Interdiscip. Top..

[50] Haus H., Shank C. (1976). Antisymmetric taper of distributed feedback lasers. IEEE J. Quantum Electron..

[51] Soda H., Kotaki Y., Sudo H., Ishikawa H., Yamakoshi S., Imai H. (1987). Stability in single longitudinal mode operation in GaInAsP/InP phase-adjusted DFB lasers. IEEE J. Quantum Electron..

[52] Agrawal G.P., Geusic J.E., Anthony P.J. (1988). Distributed feedback lasers with multiple phase-shift regions. Appl. Phys. Lett..

[53] Okai M., Chinone N., Taira H., Harada T. (1989). Corrugation-pitch-modulated phase-shifted DFB laser. IEEE Photonics Technol. Lett..

[54] Kulishov M., Kress B., Slavík R. (2013). Resonant cavities based on Parity-Time-symmetric diffractive gratings. Opt. Express.

[55] Zhu Y., Zhao Y., Fan J., Zhu L. (2016). Modal gain analysis of parity-time-symmetric distri buted feedback lasers. IEEE J. Sel. Top. Quantum Electron..

[56] Ke C., Li X., Xi Y. (2017). Parity-time symmetric complex-coupled distributed feedback laser with excellent immunity to external optical feedback. AIP Adv..

[57] Benisty H., Brac de la Perrière V.B., Ramdane A., Lupu A. (2021). Parity-time symmetric gratings in 1550 nm distributed-feedback laser diodes: Insight on device design rules. J. Opt. Soc. Am. B.

[58] Song Q., Li J., Sun W., Zhang N., Liu S., Li M., Xiao S. (2015). The combination of directional outputs and single-mode operation in circular microdisk with broken PT symmetry. Opt. Express.

[59] Gu J., Xi X., Ma J., Yu Z., Sun X. (2016). Parity–time-symmetric circular Bragg lasers: A proposal and analysis. Sci. Rep..

[60] Zhu Y., Zhao Y., Zhu L. (2017). Modal discrimination in parity-time-symmetric single microring lasers. IEEE Photonics J..

[61] Gu Z., Zhang N., Lyu Q., Li M., Xiao S., Song Q. (2016). Experimental demonstration of PT-symmetric stripe lasers. Laser Photonics Rev..

[62] Wong Z.J., Xu Y.-L., Kim J., O’Brien K., Wang Y., Feng L., Zhang X. (2016). Lasing and anti-lasing in a single cavity. Nat. Photonics.

[63] Peng R., Li Y., Huang W. (2018). A single-mode laser based on parity-time-symmetry structured vertical Bragg reflection waveguide. J. Lightwave Technol..

[64] Peng R., Sun S. (2022). A Bragg reflection waveguide laser operating at an exceptional point. Appl. Phys. Express.

[65] Wang L., Cheng X., Zhang X., Yu J., Xia M., Li C., Lin X., Liu F., Jin C. (2022). PT symmetric single-mode line-defect photonic crystal lasers with asymmetric loss design. Opt. Lett..

[66] Miao P., Zhang Z., Sun J., Walasik W., Longhi S., Litchinitser N.M., Feng L. (2016). Orbital angular momentum microlaser. Science.

[67] Zhang Z., Qiao X., Midya B., Liu K., Sun J., Wu T., Liu W., Agarwal R., Jornet J.M., Longhi S. (2020). Tunable topological charge vortex microlaser. Science.

[68] Phang S., Vukovic A., Creagh S.C., Sewell P.D., Gradoni G., Benson T.M. (2016). Localized Single Frequency Lasing States in a Finite Parity-Time Symmetric Resonator Chain. Sci. Rep..

[69] Song W., Sun W., Chen C., Song Q., Xiao S., Zhu S., Li T. (2019). Breakup and Recovery of Topological Zero Modes in Finite Non-Hermitian Optical Lattices. Phys. Rev. Lett..

[70] Peng R., Li Y., Huang W. (2019). High-Power Edge-Emitting Laser Based on a Parity-Time-Structured Bragg Reflection Waveguide. Appl. Opt..

[71] Brandstetter M., Liertzer M., Deutsch C., Klang P., Schöberl J., Türeci H.E., Strasser G., Unterrainer K., Rotter S. (2014). Reversing the pump dependence of a laser at an exceptional point. Nat. Commun..

[72] Peng B., Özdemir S.K., Rotter S., Yilmaz H., Liertzer M., Monifi F., Bender C.M., Nori F., Yang L. (2014). Loss-induced suppression and revival of lasing. Science.

[73] Rüter C.E., Makris K.G., El-Ganainy R., Christodoulides D.N., Segev M., Kip D. (2010). Observation of parity–time symmetry in optics. Nat. Phys..

[74] Miri M.A., LiKamWa P., Christodoulides D.N. (2012). Large area single-mode parity–time-symmetric laser amplifiers. Opt. Lett..

[75] Yao R., Lee C., Podolskiy V., Guo W. (2019). Electrically injected parity time–symmetric single transverse–mode lasers. Laser Photonics Rev..

[76] Fu T., Wang Y., Zhou X., Du F., Fan J., Wang X., Chen J., Qi A., Zheng W. PT-symmetric double ridge semiconductor lasers emitting at 980 nm. Proceedings of the Conference on Lasers and Electro-Optics.

[77] Yang S., Luan J., Han Y., Zhang R., Tian Q., He P., Liu D., Zhang M. (2021). High-Speed and Single-Mode FP Laser Based on Parity-Time Symmetry. arXiv.

[78] Wang X., Li J., Fu T., Chen J., Dai Y., Han R., Wang Y., Zheng W. (2022). Electrically injected double-taper semiconductor laser based on parity-time symmetry. Electron. Lett..

[79] Şeker E., Olyaeefar B., Dadashi K., Şengül S., Teimourpour M.H., El-Ganainy R., Demir A. (2023). Single-mode quasi PT-symmetric laser with high power emission. Light Sci. Appl..

[80] Wang S., Lv Z., Yang Q., Wang S., Chai H., Meng L., Lu D., Ji C., Yang X., Yang T. (2023). High-Power, Narrow-Linewidth, and Low-Noise Quantum Dot Distributed Feedback Lasers. Laser Photonics Rev..

[81] Sun Y., Xu Y., Zhang J., Chen F., Liu J., Liu S., Lu Q., Zhuo N., Wang L., Liu F. (2023). High-Power Distributed Feedback Lasers Based on InP Corrugated Sidewalls at *λ*∼2 μm. Photon. Res..

[82] Yang S., Han Y., Zhang R., Tian Q., He P., Liu D., Zhang M. (2023). Single transverse-mode semiconductor laser arrays by parity-time symmetry breaking. IEEE Photonics Technol. Lett..

[83] Hodaei H., Miri M.A., Heinrich M., Christodoulides D.N., Khajavikhan M. (2014). Parity-time–symmetric microring lasers. Science.

[84] Hodaei H., Miri M.A., Hassan A.U., Hayenga W.E., Heinrich M., Christodoulides D.N., Khajavikhan M. (2015). Parity-time-symmetric coupled microring lasers operating around an exceptional point. Opt. Lett..

[85] Liu W., Li M., Guzzon R.S., Norberg E.J., Parker J.S., Lu M., Coldren L.A., Yao J. (2017). An integrated parity-time symmetric wavelength-tunable single-mode microring laser. Nat. Commun..

[86] Hayenga W.E., Parto M., Garcia-Gracia H., Sanchez-Cristobal E., Hodaei H., Likamwa P., Christodoulides D.N., Khajavikhan M. (2017). Towards electrically injected parity-time-symmetric microring lasers. Proceedings of the IEEE Photonics Conference (IPC).

[87] Hayenga W.E., Garcia-Gracia H., Sanchez Cristobal E., Parto M., Hodaei H., LiKamWa P., Christodoulides D.N., Khajavikhan M. (2020). Electrically pumped microring parity-time-symmetric lasers. Proc. IEEE.

[88] Ting F., Yu W., Xue W., Jing C., Xu Z., Wan Z. (2021). Microstructure Lasers Based on Parity-Time Symmetry and Supersymmetry. Chin. J. Lasers.

